# The Functional Domain of GCS1-Based Gamete Fusion Resides in the Amino Terminus in Plant and Parasite Species

**DOI:** 10.1371/journal.pone.0015957

**Published:** 2010-12-31

**Authors:** Toshiyuki Mori, Makoto Hirai, Tsuneyoshi Kuroiwa, Shin-ya Miyagishima

**Affiliations:** 1 Miyagishima Initiative Research Unit, Advanced Science Institute, RIKEN, Saitama, Japan; 2 Department of Parasitology, Graduate School of Medicine, Gunma University, Gunma, Japan; 3 Department of Life Science, College of Science, Research Information Center for Extremophile, Rikkyo University, Tokyo, Japan; Temasek Life Sciences Laboratory, Singapore

## Abstract

Fertilization is one of the most important processes in all organisms utilizing sexual reproduction. In a previous study, we succeeded in identifying a novel male gametic transmembrane protein GCS1 (GENERATIVE CELL SPECIFIC 1), also called HAP2 (HAPLESS 2) in the male-sterile *Arabidopsis thaliana* mutants, as a factor critical to gamete fusion in flowering plants. Interestingly, GCS1 is highly conserved among various eukaryotes covering plants, protists and invertebrates. Of these organisms, *Chlamydomonas* (green alga) and *Plasmodium* (malaria parasite) GCS1s similarly show male gametic expression and gamete fusion function. Since it is generally believed that protein factors controlling gamete fusion have rapidly evolved and different organisms utilize species-specific gamete fusion factors, GCS1 may be an ancient fertilization factor derived from the common ancestor of those organisms above. And therefore, its molecular structure and function are important to understanding the common molecular mechanics of eukaryotic fertilization. In this study, we tried to detect the central functional domain(s) of GCS1, using complementation assay of *ArabidopsisGCS1* mutant lines expressing modified GCS1. As a result, the positively-charged C-terminal sequence of this protein is dispensable for gamete fusion, while the highly conserved N-terminal domain is critical to GCS1 function. In addition, *in vitro* fertilization assay of *Plasmodium berghei* (mouse malaria parasite) knock-in lines expressing partly truncated GCS1 showed similar results. Those findings above indicate that the extracellular N-terminus alone is sufficient for GCS1-based gamete fusion.

## Introduction

Angiosperm fertilization is comprised of certain processes, from pollination to gamete fusion [1]. Each pollen grain (male gametophyte) contains a pair of sperm cells (male gametes), and elongates a pollen tube into the pistil to deliver the sperm pair towards an ovule contained in an ovary after pollination. When the pollen tube reaches the gate of the ovule (micropyle), it releases the sperm pair into an embryo sac (female gametophyte) enclosed in the ovule wall. Female gametes, namely egg and central cells, exist close by in an embryo sac and fuse with these sperm cells to produce an embryo and an endosperm, respectively (double fertilization).

In our previous study, we succeeded in identifying the novel protein GCS1 in male generative cells isolated from *Lilium longiflorum* pollen [2]. Most angiosperm GCS1s are composed of approximately 700 amino acid residues, and are predicted to be a single-pass transmembrane protein, because of the N-terminal signal sequence and C-terminal transmembrane domain [2]–[3]. It has been found that *Arabidopsis* GCS1 is identical to HAP2, which was previously identified as a pollen tube related factor from the *hap2* phenotypes [4]. *Lilium* and *Arabidopsis* GCS1s were demonstrated to be expressed exclusively in male gametes (generative and sperm cells) and localized to the cell surface [2]–[3]. Furthermore, *Arabidopsis GCS1* mutant pollen exhibits serious male sterility in which none of the sperm cells are able to fuse with female gametes, suggesting that GCS1 is an indispensable factor for gamete fusion [2]–[3].

Surprisingly, GCS1 is highly conserved and putative orthologs have been identified in various eukaryotes, e.g., protists, amoebae and invertebrates [2]–[3], [5]–[7]. In *Plasmodium berghei* (a rodent malaria parasite) and *Chlamydomonas reinhardtii* (a green alga), it has been shown that their GCS1 is similarly expressed in the male gamete and functions in gamete fusion [5]–[6]. The *GCS1*-knockout *Chlamydomonas* male cannot perform gamete fusion, but does achieve attachment based on FUS1, which is a transmembrane protein expressed exclusively in the female gamete [8]–[9], and therefore GCS1 is expected to function in membrane fusion or in events immediately after attachment [6]. Furthermore, a recent paper reported testis-specific *GCS1* expression in the hydra (a cnidarian), implying that animal GCS1s function in a similar manner [7].

Since GCS1 possesses no known functional protein functional domains, the molecular structure and central domain(s) for gamete fusion are important issues [10]–[11]. A recent study on *Chlamydomonas* GCS1 revealed GCS1 to be a glycoprotein in which two types of N-glycosylation occur, and a rapid degradation of GCS1 molecules is triggered by gamete membrane fusion so as to prevent polygamy [12]. Furthermore, Wong *et al.* investigated the molecular importance of N- and C-terminal sequences for the GCS1 transmembrane domain, using partially-modified *AtGCS1* constructs [11]. In their study, entire deletion of either terminus leads to failure in complementation of the *AtGCS1* mutation, roughly suggesting that both termini are required for the GCS1 function of gamete fusion. In addition, they indicated that the positively-charged histidine rich domains are indispensable for normal double fertilization, since reduction of the positive charge causes reduced fertility and an occasional single fertilization, where only one sperm cell fuses with the egg or central cell in an ovule [11].

In the present study, an effort was made to obtain more detailed characteristics of GCS1 structure and function using partially-modified *AtGCS1* constructs, which are based on green fluorescence protein (GFP) insertion targeted to certain characteristic AtGCS1 sequence regions. To ensure the conservation of these GCS1 characteristics, similar constructs were also produced in PbGCS1. We report that the gamete-fusion functional GCS1 domain(s) is generally in the N-terminus and the function is drastically impaired even when the N-terminus is split from the gamete membrane.

## Results

### Construction of Modified AtGCS1s

**Figure 1:**
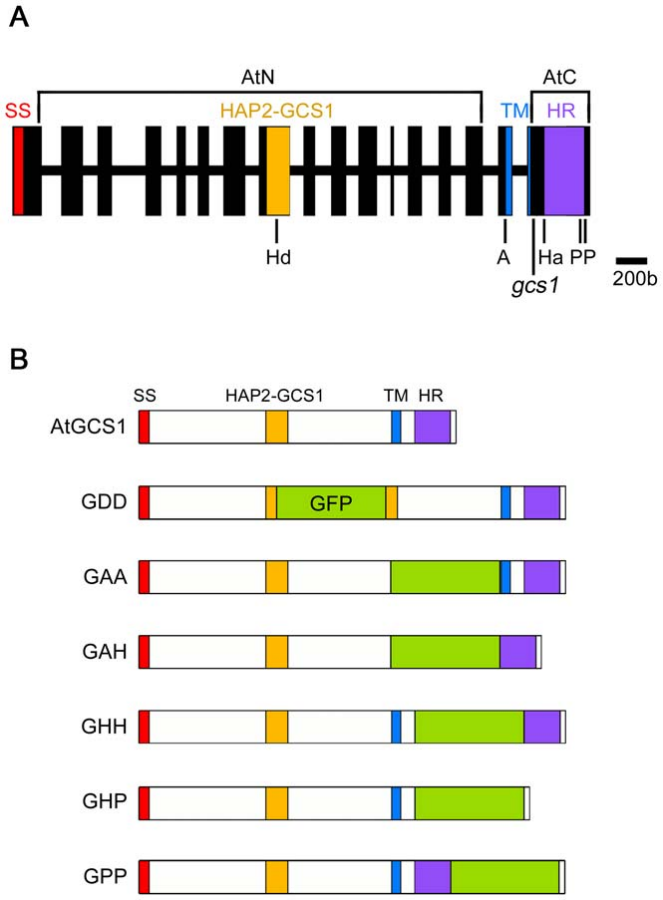
Construction of *mAtGCS1*s. (A) The characteristic structures of AtGCS1. The coding sequence of *AtGCS1* gene is composed of 17 exons (vertical bars) and 16 introns (horizonal lines) [3]. The exons coding the signal sequence (SS), HAP2-GCS1 domain, transmembrane domain (TM) and histidine-rich domain (HR) are differently colored. SS, HAP2-GCS1 and TM sequences correspond with those of previous study by von Besser *et al* [3]. HR region ranges from the head of H1 to the end of H3 in a previous study by Wong *et al* [11]. The T-DNA insert in the *AtGCS1* mutant is indicated (*gcs1*). N- and C-terminal regions (AtN and AtC respectively), which were deleted in the previous study by Wong *et al* [11], are represented. The endogenous restriction enzyme sites used for *mAtGCS1* construction are indicated with abbreviations; *Hind*III (Hd), *Afl*II (A), *Hpa*I (H), *Pml*I (P). (B) The structure of mAtGCS1 products. The top bar is the normal *Arabidopsis* GCS1 protein structure (AtGCS1). The other bars indicate mAtGCS1 protein structures with a GFP insertion. In the *GDD*, *GAA*, *GAH*, *GHH*, *GHP* and *GPP* constructs, the *GFP* cDNA insert is located in their Hd, A, A-Ha, Ha, Ha-P and P-P sites respectively.

**Figure 2:**
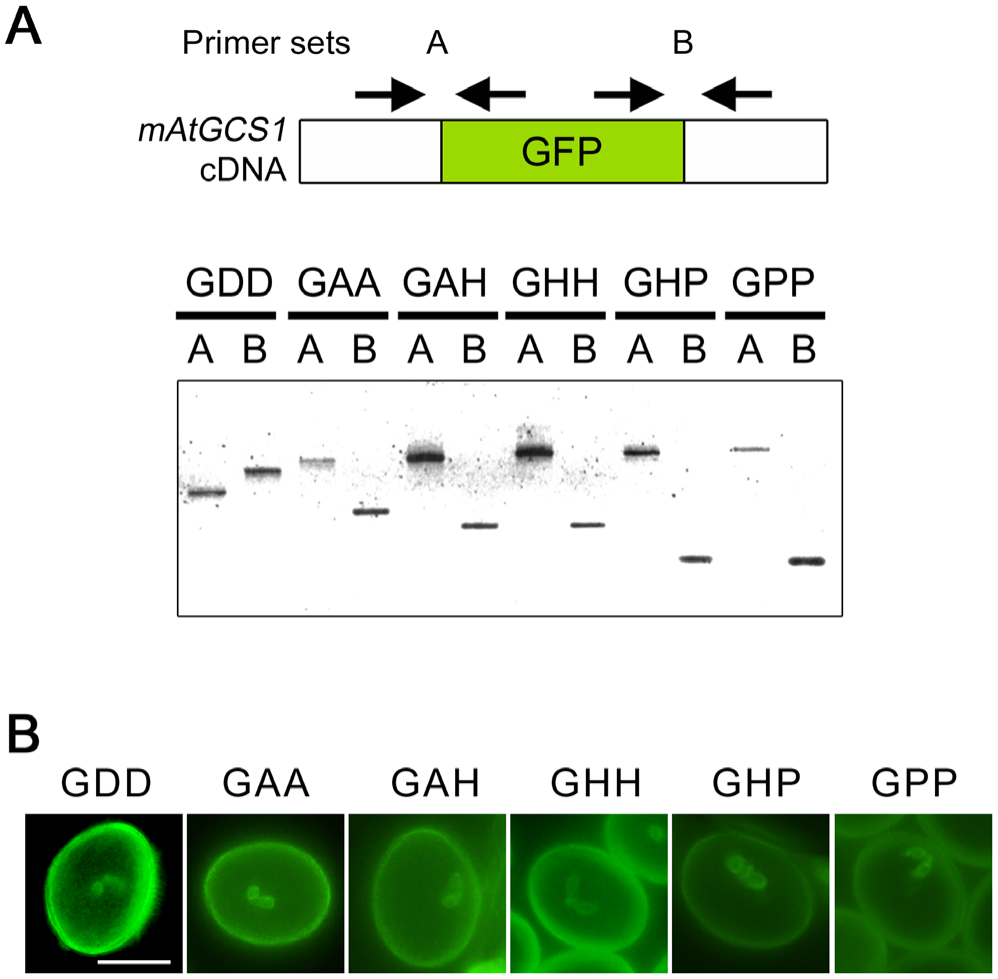
Normal expression of mAtGCS1s. (A) RT-PCR assays on the *mAtGCS1* transcripts. Total RNA was extracted from the flowers of all of the transformants, reverse-transcribed, and amplified with 2 sets of primers (primer sets A and B). The left side primer in A and the right side primer in B (i.e. the *AtGCS1* gene-specific primers) were designed from the first and last *AtGCS1* exons, respectively. Each PCR product was cloned and sequenced. (B) Protein expression of *mAtGCS1* constructs. A pair of GFP signals was detected in the pollen grains of all of the *mAtGCS1* transformants.

As previously described, AtGCS1 is composed of an N-terminus signal sequence (SS), which probably leads to its cell membrane localization, a long body sequence containing the HAP2-GCS1 domain that is highly conserved among GCS1-possessing organisms, a hydrophobic transmembrane (TM) domain and a highly basic C-terminal histidine-rich (HR) sequence (Figure 1A) [2]–[3], [11]. Unlike the previous study, in which long sequences of AtGCS1 were deleted or exchanged mainly [11], we used a different strategy that partly disrupts characteristic AtGCS1 domains with a fluorescence tag insert so that we could see the importance of each domain, and normal expression of the modified AtGCS1s as well. We first disrupted, separated, or exchanged *AtGCS1* domains described above with the *GFP* cDNA insertion at appropriate restriction-enzyme-recognizing sites. As a result, 6 modified *AtGCS1* (*mAtGCS1*) constructs were obtained (designated as *GDD*, *GAA*, *GAH*, *GHH*, *GHP* and *GPP*). The products expected from these constructs are shown in Figure 1B. In GDD, the HAP2-GCS1 domain has been disrupted. The GFP insert in GAA and GHH separates the entire N-terminal AtGCS1 sequence and HR from TM, respectively. GAH and GHP have lost TM and HR, respectively. In GPP, HR is followed by the GFP insert. These constructs were produced on the basis of a genomic *AtGCS1* clone, which was successfully used in *GCS1* knockout rescue in a previous study [2], and introduced into heterozygous *Arabidopsis GCS1* mutant (+/*gcs1*) plants to assess which of the constructs maintains or loses function, namely which domain(s) is critical to gamete fusion. The +/*gcs1* line was used in our previous study and the *gcs1* pollen is completely male-sterile [2]. Normal transcription and translation of each construct were confirmed in reverse transcription (RT)-PCR assays and by pollen observation, respectively (Figures 2A and 2B). From all results above, all constructs proved to work desirably and give no notable defects to the pollen development.

### Complement Assay of the +/*gcs1* Plants with the *mAtGCS1* Constructs

**Figure 3:**
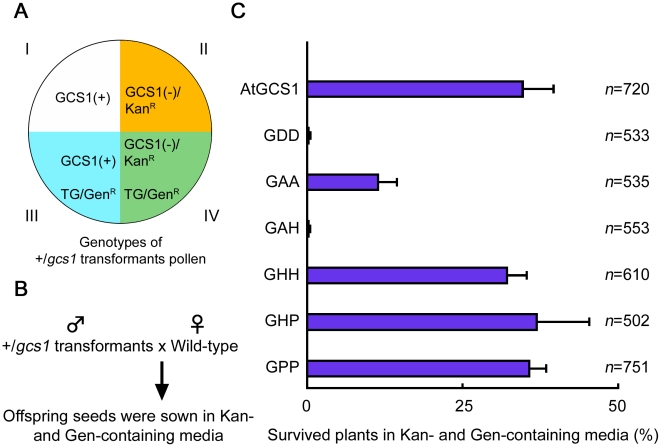
Complementation of *gcs1* sperm by introduction of *mAtGCS1* constructs. (A) The breakdown of the pollen genotypes in the +/*gcs1* transformants hemizygously expressing the *AtGCS1* or *mAtGCS1* transgene (TG) (+/*gcs1^AtGCS1^* or +/*gcs1^GDD^* ^, *GAA*, *GAH*, *GHH*, *GHP* and *GPP*^). Their pollen genotypes are dividable into 4 groups, i.e. wild-type (GCS1(+)) pollen groups without (I) and with TG (III), and those of *gcs1* (GCS1(−)) pollen groups (II and IV respectively). GCS1(−) and TG are linked with the kanamycin- (*Kan^R^*) and gentamycin-resistance (*Gen^R^*) genes respectively. (B) The assessment strategy of *gcs1* complementation. The *gcs1* complementation with each TG was decided based on the survival of offspring plants derived from the transformant pollen and wild-type females in Kan- and Gen-containing media. (C) The results on the *gcs1* complementation assay. For each TG, 3 independent transformant lines were used as pollen donors, respectively. The surviving and dead offspring plants were both counted in Kan- and Gen-containing media.

When an *mAtGCS1* construct is hemizygously introduced into +/*gcs1* plants, the pollen from the transformants are categorizable into 4 genotype groups, namely wild type pollen (I), *gcs1* pollen (II), *mAtGCS1*-possessing pollen (III) and *mAtGCS1*-possessing *gcs1* pollen (IV) (Figure. 3A). The T-DNA insert in the *gcs1* pollen is linked with the kanamycin-resistance gene (*Kan^R^*), while all of the *mAtGCS1* constructs are linked with the gentamycin-resistance gene (*Gen^R^*). Of these groups, the pollen from groups I and III is evidently fertile because of normal *GCS1* expression. On the other hand, the group II pollen is infertile because of the *GCS1* mutation, and therefore, *Kan^R^* is never inherited by the offspring plants paternally. However, if an *mAtGCS1* construct complements the *GCS1* mutation, both *Kan^R^* and *Gen^R^* in group IV should be inherited by the offspring plants paternally. In this case, one-third (∼33.3%) of the offspring should survive in the Kan- and Gen-containing media, while the remaining ones (∼66.7%) should not be alive, because they are from groups I and III in which the wild-type plants were pollinated with the transformant pollen. Based on this expected scenario, wild type females were pollinated with pollen from each of the *mAtGCS1* transformants, and the resulting offspring seeds were sown in Kan- and Gen-containing media to assess which construct complements the *GCS1* mutation (Figure 3B). As a positive control, +/*gcs1^AtGCS1^* lines, in which a wild-type genomic *AtGCS1* clone linked with Gen^R^ (*AtGCS1* construct) is hemizygously expressed, were used. As a result, when the +/*gcs1^AtGCS1^* pollen was used, approximately 33.3% of the offspring plants survived in the media (chi-square, P>0.5), indicating that complementation by the construct was almost completely successful (Figure 3C, the top bar chart). On the other hand, almost all of the plants from the +/*gcs1^GDD^* and +/*gcs1^GAH^* pollen died, while that of the +/*gcs1^GAA^* pollen exhibited weak complementation (Figure 3C). The difference between the results for +/*gcs1^GAA^* and +/*gcs1^AtGCS1^* is statistically significant (chi-square, P<0.01). Furthermore, the results for the +/*gcs1^GHH^*, +/*gcs1^GHP^* and +/*gcs1^GPP^* pollen displayed obvious complementation and were comparable with to +/*gcs1^AtGCS1^* (chi-square, P>0.5) (Figure 3C). At the same time, these results for *GHH*, *GHP* and *GPP* constructs indicate that their C-terminal GFP inserts did not dominantly affect the fertility of wild-type pollen.

### Sperm Activity in +/*gcs1^GAA^* Plants

**Figure 4:**
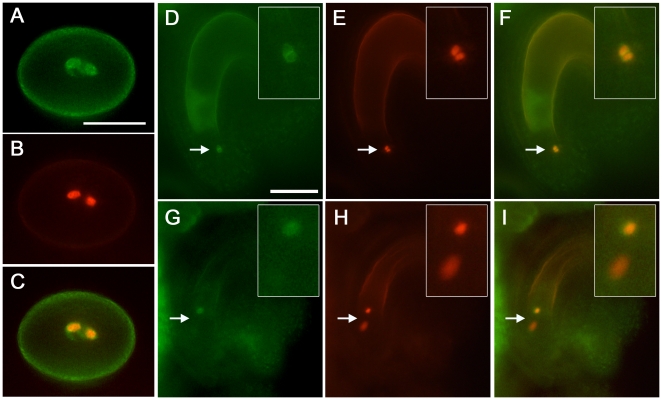
Observation of +/*gcs1^GAA^* sperm cells in ovules. (A–C) Production of a +/*gcs1^GAA^* line expressing a sperm nucleus marker. A pair of sperm cells expresses both GFP (A) and RFP (B) signals from the *GAA* and *pHTR10::HTR10-RFP* constructs, respectively. (A and B) are an identical field pair and merged in (C). (D–I) Observation of unfertilized sperm cells in the +/*gcs1^GAA^* line. Self-pollinated +/*gcs1^GAA^* pistils at 24 HAP were disassembled and their ovules were observed. A GFP signal pair was occasionally detected in the ovules (D). Such signal pairs also exhibited the RFP signal pair, confirming that the GFP signals are indeed from the sperm pair (E). On the other hand, some sperm pairs showed single fertilization (G and H). In (G and H), the top sperm cell persists unfertilized, while the bottom one, showing a dispersed chromatin that has ongoing karyogamy with an egg cell, is in the process of losing its GFP signal. The insert in (D–I) is magnification of the area indicated by the arrow in each image. (D and E) and (G and H) are identical field pairs and merged in (F and I) respectively. Scale bars, 10 µm (A); 25 µm (D).

**Figure 5:**
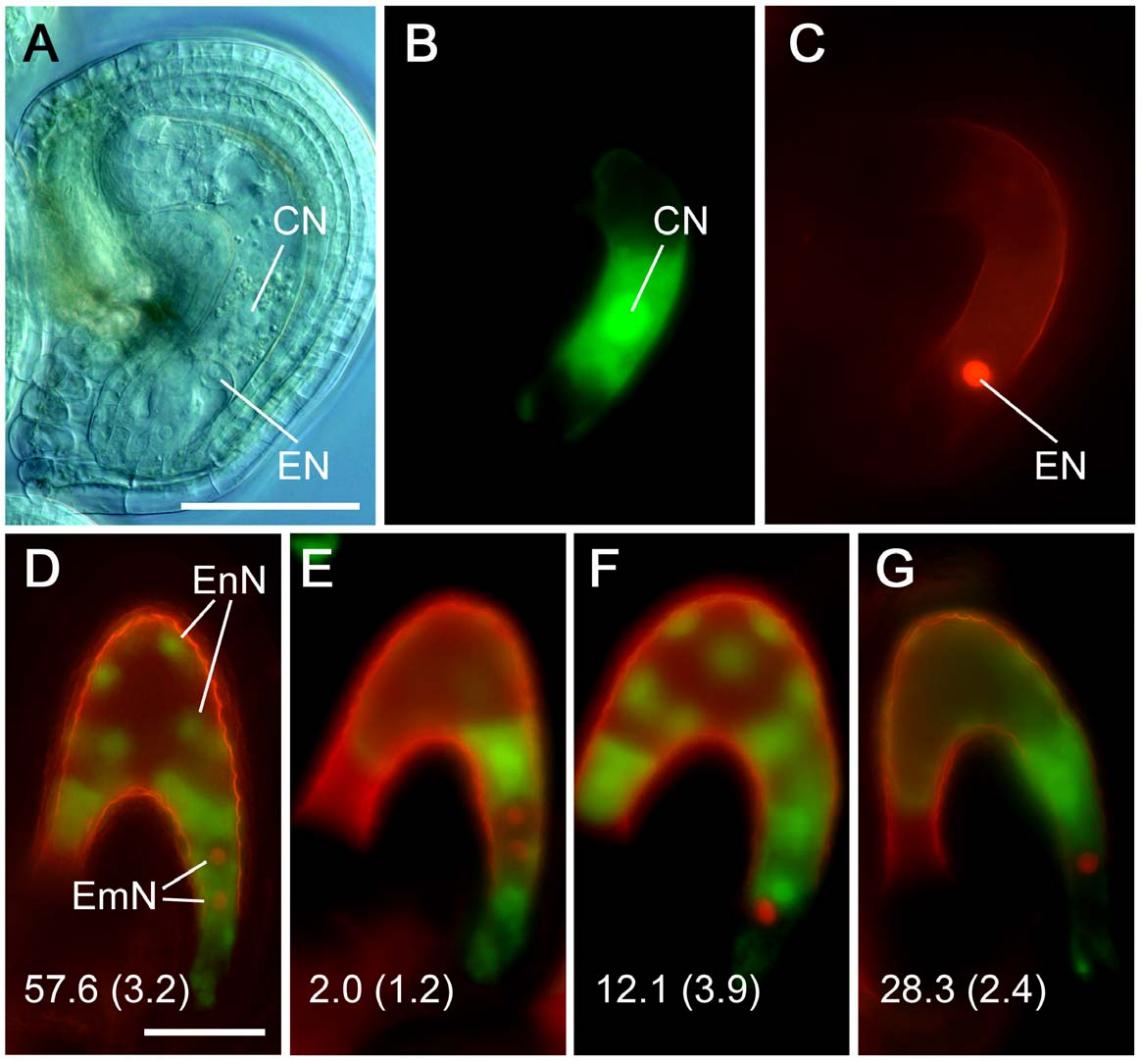
Observation of self fertilization products derived from *gcs1^GAA^* sperm cells. (A–C) Preparation of the *Arabidopsis* female marker line. In ovules of this line, central and egg cells express ΔFWA-GFP and H2B-RFP driven by the pFWA and pEC1 promoters, respectively (B and C respectively). (D–G) This marker line was pollinated with pollen from the *+/gcs1^+/GAA^* line, where GAA is homozygously expressed. When normal double fertilization by wild-type- and complemented *gcs1* sperm cells takes place, both embryogenesis and endosperm development are observed as their nuclei proliferate (D). On the other hand, development of the embryo or endosperm alone, resulting from delayed gamete fusion, was occasionally observed (E and F respectively). Extensively delayed double fertilization leads to there being no seed development (G). The percentage of each fertilization type observed in 3 plants is shown with the s.d. in the parenthesis (*n* = 279) (D–G). EnN, endosperm nuclei; EmN, embryo nuclei. Scale bars, 50 µm.

The weak *gcs1* complementation by GAA led to the expectation that the +/*gcs1^GAA^* sperm cells occasionally fail to fuse. To observe the sperm behavior in an ovule, we produced a +/*gcs1^GAA^* line whose sperm cells express HTR10-RFP fusion as a sperm nucleus marker [13], in addition to the GFP signal from GAA (Figures 4A–C). When isolated ovules were observed at 24 h after pollination (HAP), a pair of unfertilized sperm cells was occasionally detected in an ovule as a GFP signal pair (Figure 4D). Most of these sperm cells also expressed RFP signals and were detected in the vicinity of the border between the egg and central cells, indicating that GAA is less functional than the other *mAtGCS1* constructs which are successful in complementation, such as *GHH*, *GHP* and *GPP* (Figures S1, 4E and 4F). Less frequently, it was observed that one sperm cell was fusing with an egg cell, while the remaining one of the pair was left unfertilized and retained the GFP signal (Figures 4G–I). To trace fertilization products derived from such incomplete gamete fusion, an *Arabidopsis* line that express H2B-RFP and ΔFWA-GFP as egg and central cell division marker respectively [14]–[16], was produced and pollinated with +/*gcs1^+/GAA^* line pollen, in which sperm cells homozygously express GAA (Figures 5A–C). As a result, when the ovules at 30 HAP were observed, single fertilization products were occasionally detected (Figures 5D–G). Taken together, we conclude that GAA is not stably functional and the GFP insert separating the entire N-terminus from TM might have given an altered feature to the GCS1 molecule.

### Conservation of GCS1 functional domains

**Figure 6:**
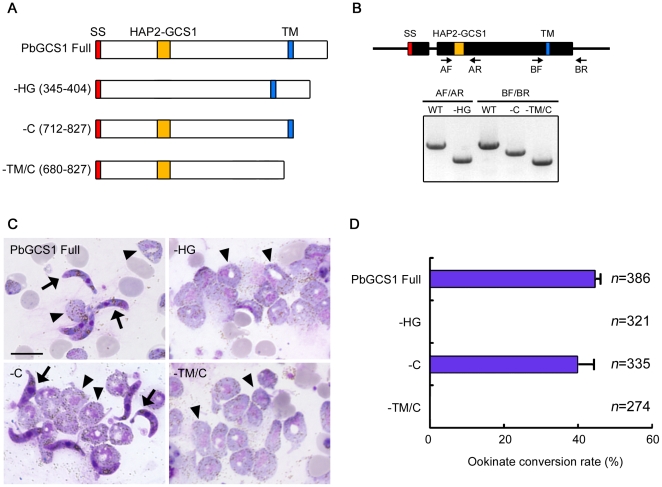
Characterization of PbGCS1 domains using *mPbGCS1* constructs. (A) The structure of the modified PbGCS1 (mPbGCS1) products. The top bar is the normal PbGCS1 protein structure (PbGCS1 Full). The signal sequence (SS), HAP2-GCS1 and transmembrane domains (TM) are differently colored. The numbers in parentheses indicate the number of the deleted amino acids of mPbGCS1. –HG, –C and –TM/C indicate the deletion of the HAP2-GCS1, C terminal, and TM and C terminal domains, respectively. (B) RT-PCR assay of the *mPbGCS1* transcripts. Total RNA was extracted from transgenic parasites, reverse-transcribed, and amplified with 2 sets of primers (primer sets A and B). The primer set A was designed to confirm the successful elimination of HAP2-GCS1 domain in –HG parasite, while the primer set B was designed for the C and TM/C domain deletion in the –C and –TM/C parasites. Each PCR product was cloned and sequenced. (C) Ookinetes and unfertilized females of the transgenic parasites. Each transgenic parasite was cultured for 16 h and stained with Giemsa. Arrowheads and arrows indicate female gametes and ookinates, respectively. Scale bar, 10 µm. (D) Fertility of transgenic parasites. The bars represent the rate of females that were fertilized by males and resulting in ookinetes. For each parasite line, 3 independent clones were assayed for ookinate formation (triplicate/clone). Error bars represent mean ± s.d.

To confirm whether the importance of the GCS1 domain is generally conserved in other organisms, a similar GCS1 modification assay was performed in *P. berghei* GCS1 (PbGCS1). In this assay, we prepared 3 partially-modified *PbGCS1* constructs, designated –*HG*, –*C* and –*TM/C*, besides the normal *PbGCS1* construct (*PbGCS1 Full*). The expected product from each construct is shown in Figure 6A. In the –HG, –C and –TM/C peptides, the HAP2-GCS1 domain, the entire C-terminal region from TM, and a sequence covering the TM domain and the entire C-terminal region, were respectively deleted. Each construct was introduced into *P. berghei* parasites by double crossover homologous recombination, by which the endogenous *PbGCS1* gene is replaced with each construct. Normal expression of the transgene in these knock-in (KI) parasites was confirmed with RT-PCR assay (Figure 6B).

Since the *in vitro* fertilization assay has been established in rodent malaria parasites [17], we used it to assess the male fertility of each KI parasite, based on the appearance of ookinates, zygotic cells resulting from successful gamete fusion. As a result, obvious ookinate conversion was frequently detected in –C KI-parasites with similar efficiency to that of PbGCS1 Full KI parasites (chi-square, P>0.5). This result indicates that the homologous recombination itself does not affect the male fertility and that the C-terminus of PbGCS1 less contributes to gamete fusion (Figures 6C and 6D). On the other hand, no ookinates were detected in –HG- and –TM/C-KI parasites, suggesting that the highly conserved N-terminal domain and TM of PbGCS1 are similarly critical to parasite gamete fusion (Figures 6C and 6D). We, therefore, conclude that the domain-deleting strategy is also applicable to investigation of molecular GCS1 functions and the results are consistent with those of AtGCS1.

## Discussion

### The C-terminus of GCS1 is Dispensable for Gamete Fusion

In a previous study, we showed that angiosperm GCS1 is a membrane-associated protein that is localized in the male gamete membrane [2]. Consistent with this, AtGCS1 without TM (GAH) exhibited impaired GCS1 function, suggesting that the TM domain is required for membrane localization of GCS1 (Figure 3C). A similar result was also obtained in –TM/C KI parasites, in which GCS1 lacks both TM- and entire C-terminal-regions (Figure 6). Since –C KI parasites, in which GCS1 lacks only the entire C-terminus, exhibited normal fertility, its TM is similarly critical to GCS1 localization.

Next question was which of the N- and C-termini to the TM is critical for the gamete fusion function of GCS1. Wong *et al.* recently reported 3 major histidine-rich regions (H1-3) in the C-terminus of AtGCS1, and demonstrated that deletion of the C-terminal sequence covering them or an exchange of their positively charged residues, resulted in an obvious reduction of GCS1 function [11]. However, our data did not show any functional deficiency in GHP, the H1-3 regions of which were all exchanged with the GFP sequence (Figures 1B and 3C). Since the GFP insert has no positive charge corresponding to the original histidine-rich regions, the hypothesis of the C-terminus contribution would appear to be controversial. In addition, the C-terminus-deleted GCS1 was also functional in gamete fusion in the malaria parasite (Figure 6). This indicates that the C-terminus contribution to gamete fusion is at least not conserved as a general GCS1 feature. As in the case of GHP, functional deficiency was not observed in either GHH or GPP, in which the GFP insert is flanked by HR, indicating that the GFP structure itself did not inflict any damage on the C-terminus. These markers are useful to track GCS1 behavior during gamete fusion, and some are currently being used for the purpose of a live imaging of *Arabidopsis* gamete fusion (unpublished data). As an explanation for our results conflicting with the hypothetical C-terminus contribution, C-terminal domain(s) may be associated with functional localization and/or stability of AtGCS1 molecules on the sperm membrane. Indeed, the functional GHP keeps endogenous 34 amino acids between TM and the GFP insert (Figure 1B), while one of their C-terminus-deleted AtGCS1s, in which 13 amino acids following TM were kept, was not functional [11]. This fact may suggest that the 34 residues are critical to precise AtGCS1 localization, even though this hypothesis is not applicable to the *–C* construct of PbGCS1.

### The N-terminus of GCS1 Contains Functional Gamete Fusion Domain(s)

The HAP2-GCS1 domain was recently determined to be made up of highly conserved residues [2], [11]. In particular, cysteine distribution in the domain is conserved in all of the GCS1-possessing eukaryotes known to date, suggesting that it serves as an indispensable structure for gamete fusion. Besides the C-terminus-modified *AtGCS1* constructs, we produced two N-terminus constructs as well, namely *GDD* and *GAA*. In GDD, the GFP insert has resulted in a change in the cysteine residue positions in the HAP2-GCS1 domain, resulting in drastically reduced GCS1 function (Figure 3C). Also in PbGCS1, the deletion of the HAP2-GCS1 domain shows that it is vital for its function (Figures 6C and 6D). On the other hand, GAA, in which the entire N-terminal sequence is separated from TM by the GFP insert, did retain some GCS1 function. There are no cysteine residues between the GFP insert and TM in GAA, and therefore, the spatial N-terminal structure might be, at least partly, maintained and functional, even though the distance between the N-terminus and TM is extensive. Besides, a prediction of GCS1 orientation based on Hidden Markof Modeling and a gamete fusion blocking with N-terminus recognizing antibodies suggest that the N-terminus may be extracellular [11], [18]. Taken together, the central functional domain(s) of GCS1 probably resides in conserved N-terminal regions, such as the HAP2-GCS1 domain, and the putative extracellular N-terminus alone is sufficient for gamete fusion.

### Incomplete Fertilization in *gcs1^GAA^* Sperm Cells

To date, incomplete double fertilization (i.e. single fertilization), where only one sperm cell fuses with a female partner cell in an ovule, has been reported in various *Arabidopsis* male gametic mutants and transgenic lines [16], [19]–[21]. In all such lines, the single fertilization occurs in both sperm-egg and sperm-central combinations, resulting in fertilization products of the embryo or the endosperm alone. Also in +/*gcs1^GAA^* plants, both fertilization patterns were observed, (Figures 5E and 5F). When ovules of +/*gcs1^GAA^* were observed at 24 HAP, sperm nuclei in karyogamy were occasionally detected (Figure 4H). Since a previous study showed that karyogamy between sperm and egg/central cells is completed within 7–8 HAP [22], such karyogamy at 24 HAP is extremely late. Since wild-type *Arabidopsis* lines homozygously expressing GAA showed no abnormalities in their seed sets (data not shown), the +/*gcs1^GAA^* phenotypes might not be attributed to neofunctionalization by *GAA* products. We, therefore, conclude that the original *gcs1* phenotype, namely no gamete fusion, was converted into delayed gamete fusion by the introduction of *GAA*. Nevertheless, the fact that some *gcs1* sperm cells were complemented by GAA indicates that slightly delayed gamete fusion is not crucial for double fertilization, while extensively delayed fertilization results in a failure of seed development (Figure 3C).

### Conclusion

GCS1 is highly conserved and putative orthologs have been identified in various eukaryotes, e.g., protists, amoebae and invertebrates, and therefore, its molecular structure and function are important to understanding the common molecular mechanics of fertilization in these organisms. In addition, understanding of malaria parasite fertilization based on GCS1 may be applicable to novel strategies attacking parasites [18], [23]. Since GCS1 is a novel transmembrane protein with no known functional domains, it is still difficult to analyze its molecular function during gamete fusion by means of studies using GCS1 alone. If new GCS1-related factors were to be identified, whether they are male or female ones, the understanding of the importance of each GCS1 domain would likely be accelerated. In the present study, we demonstrated that the N-terminus of GCS1 by itself is sufficient for gamete fusion and a highly conserved region, such as the HAP2-GCS1 domain, is critical to successful fertilization in both plants and parasties. This suggests that such a domain possesses a fundamental function indispensable for gamete fusion in GCS1-possessing organisms. Further investigations, focusing on the N-terminus by itself, may help achieve the isolation of GCS1-related fertilization factors and an understanding of the nature of gamete fusion in the future.

## Materials and Methods

### Ethics Statement

Studies with experimental animals were approved by Animal Care and Use Committee of Gunma University, and followed guidelines of this committee. The transgenic P. berghei was generated under the guidelines of the recombinant DNA experiments committee of Gunma University. The assigned ID for above experiments is 10-007.

### Sequences of Oligonucleotide Primers

ATGCS1PROF(SacI) TGAGGC*GAGCTC*AGCAGAGCACATCTTATCAATTTC;

GCS1GENF1000(KpnI), TGAGGC*GGTACC*AGCAGAGCACATCTTATCAATTTC;

ATGCS1GENR(KpnI), TGAGGC*GGTACC*GAAACCCTAACTTAATCGAATCGT;

GFPf2(HindIII), TGAGGC*AAGCTT*TCATGGTGAGCAAGGGCGAGGA;

GFPr2(HindIII), TGAGGC*AAGCTT*CCTTGTACAGCTCGTCCATGCCG;

GFPf(AflII), TGAGG*CTTAAG*CATGGTGAGCAAGGGCGA;

GFPr(AflII), TACT*CTTAAG*TTCTTGTACAGCTCGTCCAT;

GFPf(HpaI), TGAGGT*GTTAAC*ATGGTGAGCAAGGGCGA;

GFPr(HpaI), TGACTT*GTTAAC*CTTGTACAGCTCGTCCAT;

GFPf(PmlI), TAGGT*CACGTG*ATATGGTGAGCAAGGGCGA;

GFPr(PmlI), TGACTT*CACGTG*CTTGTACAGCTCGTCCAT;

Atgcs1-seqA2, GTCTTGAATCTCGCTGTTCCCAGT;

Atgcs1truer, TTAACTCTCACGTAGTCTTTGTTTCC;

GFPBDr, CGGACACGCTGAACTTGTGG;

GFPBDf, GCCCTGAGCAAAGACCCCAA.

PbGCSF(KpnI), GG*GGTACC*GATCATGTTGGAGAATAAATGGG

PbGCSR(ClaI), GG*ATCGAT*AATTAAAGAAATTATATTTATCTGTC

PbGCS3UTRF(EcoRV), GG*GATATC*AATTACATGGAATAGTATTTGCAAATTTG

PbGCS3UTRR(XbaI), GG*TCTAGA*GCCTTTTATAATATGATATTACCCAC

-HAP2/GCS1InvR, CATGGATGCATAATATTACATATACTTG

-HAP2/GCS1InvF, TATAAATACAATGGAAATCATAGTGTAG

-CInvR, AGTAGATATAAAAAGGAAAAAGAAATTTAAAAAGAATGGTAGAAG

-CInvF, TAAAATTACATGGAATAGTATTTGCAAATTTGTG

-TMR, CCCAATTAACGTTTTAACTGTATTTATATAATAGT

AF, GACATTGTCCTATTATAGATTTAATGC

AR, TGAGTAATTGTTGCAATGGAAACGGGTC

BF, CTCTTGCTCAATTGCATGC

BR, GTTGTGTTTCCTCCATCC

### Experimental Materials

The *Arabidopsis GCS1* mutant seeds were obtained as previously described. All *Arabidopsis* plants (Columbia ecotype) were grown in an incubator on a 16-light/8-h dark at 20°C. *Plasmodium berghei* (ANKA clone 2.34) was intraperitonealy injected into a Balb/c female mouse.

### Production of GCS1 constructs and *Arabidopsis* transformants

For production of the *AtGCS1* construct, an *AtGCS1* genomic clone containing its own promoter (1.0 kb) and terminator (0.4 kb) was prepared using genomic PCR with the primers ATGCS1PROF(SacI), GCS1GENF1000(KpnI) and ATGCS1GENR(KpnI), and cloned into the *pPZP221* binary vector after digestion by the appropriate restriction enzymes. For +/*gcs1^AtGCS1^* production, this construct was simply introduced into +/*gcs1* plants. For the production of *mAtGCS1* constructs (*GDD*, *GAA*, *GAH*, *GHH*, *GHP* and *GPP*), an *GFP* cDNA was amplified with the primer pairs GFPf2(HindIII) and GFPr2(HindIII), GFPf(AflII) and GFPr(AflII), GFPf(AflII) and GFPr(HpaI), GFPf(HpaI) and GFPr(HpaI), GFPf(HpaI) and GFPr(PmlI), and GFPf(PmlI) and GFPr(PmlI), respectively. These PCR products were digested and inserted into the *AtGCS1* construct as above, using the appropriate restriction enzymes. All the *Arabidopsis* T_1_ transformants were generated by *Agrobacterium*-mediated infiltration, as previously described, and selected in plate media containing 0.01% (w/v) kanamycin and 0.01% gentamycin (w/v). Regarding +/*gcs1^GAA^*, the T_2_ +/*gcs1* plants homozygously expressing GAA (+/*gcs1^+/GAA^*) were also recovered. For confirmation of the suspected transcription, total RNA samples were extracted from the flowers of each of the transformants, reverse-transcribed, and amplified with primer pairs Atgcs1-seqA2 and GFPBDr, and GFPBDf and Atgcs1truer (primer sets A and B in Figure 1C, respectively).

### The *gcs1* Complementation Assays

Emasculated wild-type pistils were pollinated with pollen from the +/*gcs1* transformants described above. The resulting offspring seeds were sown in the same media as in the transformant selection. Surviving and dead seedlings were counted in the plate media.

### Observation of Incomplete Double Fertilization in +/*gcs1^GAA^*

The +/*gcs1* plants were crossed with the transgenic *Arabidopsis* expressing HTR10-RFP (sperm nucleus marker), and the F_1_ +/*gcs1* plants hemizygously expressing the marker were obtained. The F_1_ plants were transformed with the *GAA* construct and the T_1_ +/*gcs1^GAA^* plants homozygously expressing HTR10-RFP were selected in the media as above. Their hand-pollinated pistils were disassembled at 24 HAP and the developing ovules were isolated and observed under fluorescence microscopy. The *Arabidopsis* lines, expressing H2B-RFP and ΔFWA-GFP driven by the pEC1 and pFWA promoters respectively, were crossed, and the F_2_ plants homozygously expressing both markers were recovered. The obtained plants were pollinated with +/*gcs1^+/GAA^* pollen and the fertilized ovules were observed at 30 HAP.

### Production of *mPbGCS1* Constructs and mPbGCS1-Expressing *P. berghei*

For production of the *PbGCS1 Full* construct, two *PbGCS1* gene fragments covering a region from its own promoter (0.6 kb) to terminator (1.5 kb), and only the terminator (0.6 kb), were prepared using genomic PCR with two primer sets, PbGCSF(KpnI)/PbGCSR(ClaI) and PbGCS3UTRF(EcoRV)/PbGCS3UTRR(XbaI), respectively. Each PCR fragment was cloned into each side of the selectable marker gene (*TgDHFR-ts*) in *pBS-DHFR* [24]. For production of the –HG, –C and –TM/C constructs, inverse PCR was employed to remove each domain from the *PbGCS1 Full* construct with the three primer sets, –HAP2/GCS1InvF/–HAP2/GCS1InvR, –CInvF/–CInvR, and –CInvF/–TMR, and the *PbGCS1 Full* construct as a template. The PCR products were treated with DpnI to digest the *PbGCS1 Full* template plasmid, and the 5′ end was phosphorylated and then self-ligated. The resultant constructs were sequenced and cut by KpnI and XbaI. The linearized constructs were then introduced into the parasite genome by double crossover homologous recombination so that the constructs come under the control of the endogenous *PbGCS1* promoter. The parasite transfection, pyrimethamine selection, and dilution cloning were repeated tree times for each construct, as described previously [25]. Three independent parasite clones were obtained from each of the three transfections and used for the experiment. RT-PCR was performed to confirm the successful elimination of the HAP2/GCS1, C and TM/C domains in each transgenic parasite using the two sets of primers, AF/AR and BF/BR.

### Assessment of the Fertility of mPbGCS1-expressing *P. berghei*

Thin blood films prepared from mice infected with transgenic parasites were stained with Giemsa, and then parasitemia and the female gametocyte ratio were calculated. The infected blood was mixed with fertilization medium and cultured for 16 h. The thin blood films were prepared and the ookinate ratio was calculated. The efficiency of fertilization was expressed as the ratio of females which were fertilized with males and transformed into ookinetes. The assay was repeated three times for each clone. Thus, the assay was repeated 9 times for each transgenic parasite.

## Supporting Information

Figure S1 **Precise observation of unfertilized** ***gcs1^GAA^*** **sperm cells.** (A–C) Detailed observation of an unfertilized +/*gcs1^GAA^* sperm pair. In the +/*gcs1^GAA^* line, in which the sperm nuclei are labeled with HTR10-RFP, an unfertilized sperm pair is occasionally visible in an ovule. In a visualized embryo sac under differential interference contrast (DIC) microscopy (A), such sperm pairs were detected in the vicinity of female gametes as GFP and RFP signal pairs (B and C, respectively). (A–C) are an identical field group. (D–F) are magnification of the area indicated by the arrow in (A–C), respectively, and they are merged in (G). The arrowheads indicate the sperm pairs. CN, central cell nucleus; EN, egg cell nucleus; DSY, degenerated synergid cell. Scale bars, 25 µm (A); 5 µm (D).
(TIF)
