# Supplementary figures and images for: The Functional Domain of GCS1-Based Gamete Fusion Resides in the Amino Terminus in Plant and Parasite Species
Source: PLoS One. 2010 Dec 31;5(12):e15957. doi: 10.1371/journal.pone.0015957 (PMC3013147; doi:10.1371/journal.pone.0015957)

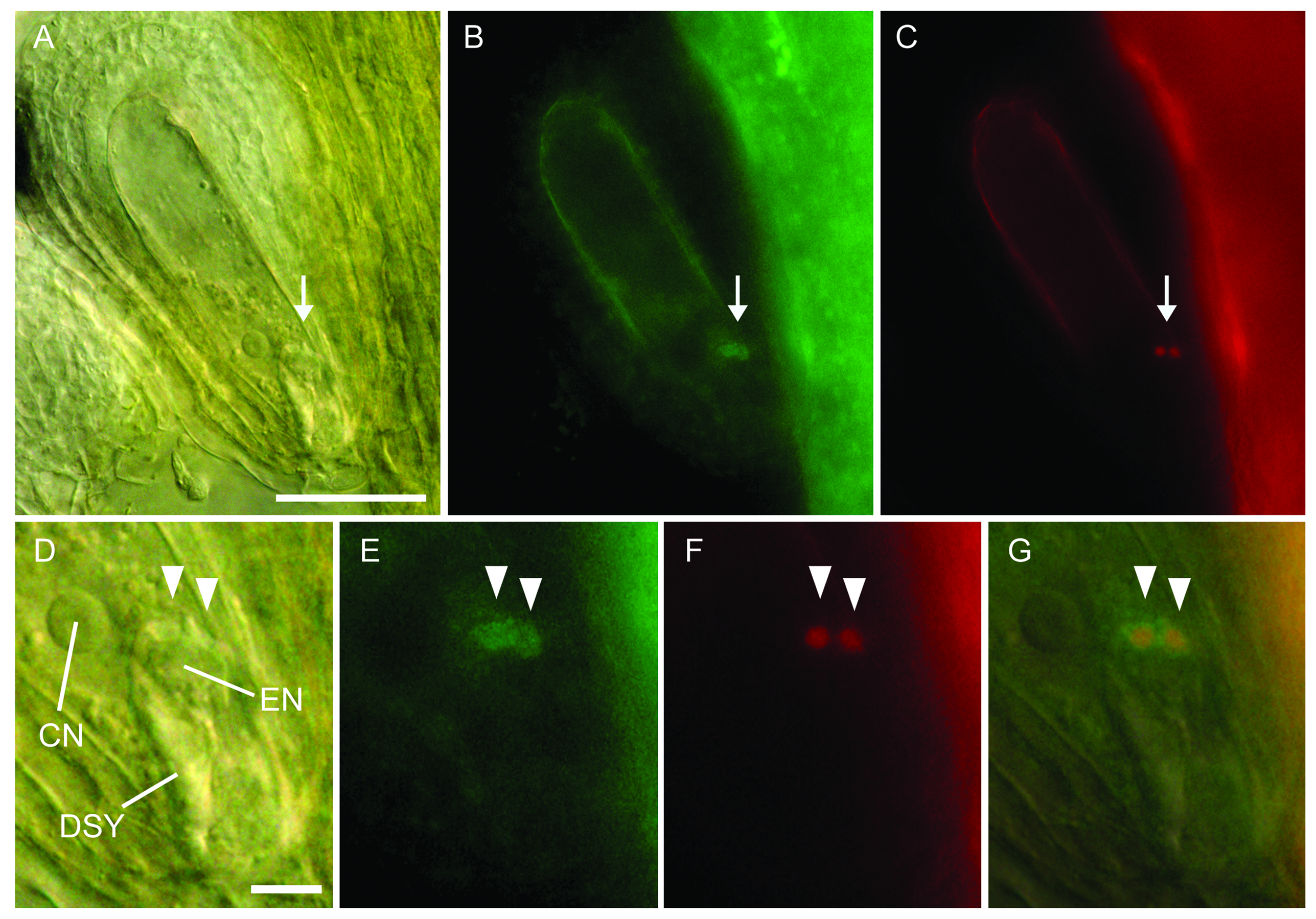

Supplement: Figure S1 — Precise observation of unfertilized gcs1GAA sperm cells. (A–C) Detailed observation of an unfertilized +/gcs1GAA sperm pair. In the +/gcs1GAA line, in which the sperm nuclei are labeled with HTR10-RFP, an unfertilized sperm pair is occasionally visible in an ovule. In a visualized embryo sac under differential interference contrast (DIC) microscopy (A), such sperm pairs were detected in the vicinity of female gametes as GFP and RFP signal pairs (B and C, respectively). (A–C) are an identical field group. (D–F) are magnification of the area indicated by the arrow in (A–C), respectively, and they are merged in (G). The arrowheads indicate the sperm pairs. CN, central cell nucleus; EN, egg cell nucleus; DSY, degenerated synergid cell. Scale bars, 25 µm (A); 5 µm (D). (TIF) [file pone.0015957.s001.tif]
